# Renal Papillary Necrosis Following Mesenteric Artery Stenting

**DOI:** 10.7759/cureus.10824

**Published:** 2020-10-06

**Authors:** Zachary A Glusman, Kenneth J Sample, Kevin S Landau, Ronald B Vigo

**Affiliations:** 1 Internal Medicine, St. George's University School of Medicine, St. George's, GRD; 2 Nephrology, Delray Medical Center, Delray Beach, USA

**Keywords:** renal papillary necrosis, renal infarction, renal thrombus, kidney, papillary necrosis, renal obstruction

## Abstract

A 62-year-old man presented with left flank pain and hematuria four days after undergoing mesenteric artery balloon angioplasty and stent placement. Imaging revealed left renal infarction with associated papillary necrosis and a thrombus in the left collecting system causing acute renal obstruction. Complete obstruction was confirmed using MAG3 Renal Scan with Lasix. A nephrostomy tube was inserted under CT guidance by interventional radiology with complete resolution of obstruction and hematuria.

## Introduction

Renal papillary necrosis (RPN) is a rare presentation of coagulative necrosis of the papilla and medullary pyramids. It is most often associated with analgesic nephropathy, sickle cell nephropathy, and diabetes mellitus complicated by urinary tract infection. However, it may also present in the setting of pyelonephritis, obstructive uropathy, hepatopathology, tuberculosis, renal transplant rejection, and some vasculitides or coagulopathies [1,2]. This potentially devastating process can lead to secondary infection of necrotic areas and may involve sloughing of the affected cells that can cause obstructive nephropathy and even complete renal failure [1].

## Case presentation

A 62-year-old Caucasian man presented with complaints of palpitations and leg pain with toe discoloration. The patient had associated dark stools and coffee ground emesis for two months prior to presentation. The patient denied hematuria, chest pain, dizziness, and shortness of breath. He had a smoking history of one pack per day for the majority of his adult life. On physical examination, there was no costovertebral angle tenderness. The patient had right first toe mottling and decreased capillary refill. The right fifth toe was cyanotic with an ulceration on the plantar surface. The dorsalis pedis pulses bilaterally were nonpalpable, and decreased tibial pedialis pulses were noted. An electrocardiogram (ECG) with no abnormalities and a chest X-ray showing normal pulmonary vasculature supported the absence of acute cardiopulmonary disease. Macroscopic urinalysis (UA) was light yellow and clear and microscopically there were 17 RBC/hpf and 5 WBC/hpf. Serum creatinine was 1.0 mg/dL with blood urea nitrogen (BUN) of 14 mg/dL. Hemoglobin (Hgb) and hematocrit (Hct) were 18.3 g/dL and 55.2%, respectively. A CT showed significant atherosclerotic disease with 50% stenosis of the infrarenal abdominal aorta and complete occlusion of both the common and external iliac arteries bilaterally. There was high-grade stenosis at the origin of the celiac axis and high-grade stenosis of the proximal 1.2 cm of the superior mesenteric artery. There was no evidence of renal involvement at that time. Vascular surgery performed balloon angioplasty with stent placement in the superior mesenteric artery through a left brachial artery cutdown. The patient was discharged two days later with aspirin and clopidogrel.

Four days later, he returned with left flank pain and gross hematuria with clots. Macroscopic UA was brown and turbid and revealed 182 RBC/hpf and 82 WBC/hpf. Based on the Acute Kidney Injury Network (AKIN) criteria, there was no evidence of acute kidney injury, with serum creatinine remaining stable at 0.9 mg/dL. BUN was 14 mg/dL, and Hgb and Hct were 13.7 g/dL and 41.8%, respectively.

**Figure 1:**
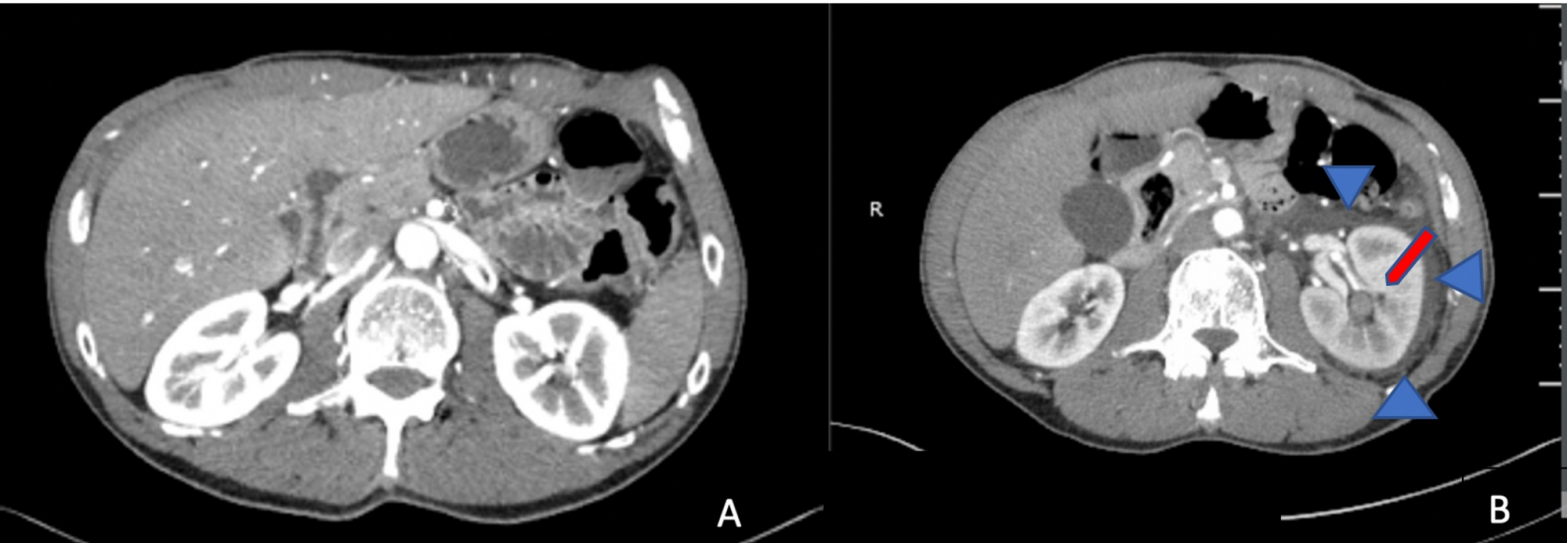
(A) CTA of the abdomen/pelvis on first admission versus (B) CTA of the abdomen/pelvis on second admission Blue arrows signify hypo-attenuated changes around the right kidney, and red arrow signifies one of the hypo-attenuated changes in the area of a renal papillae. CTA, CT angiography

**Figure 2:**
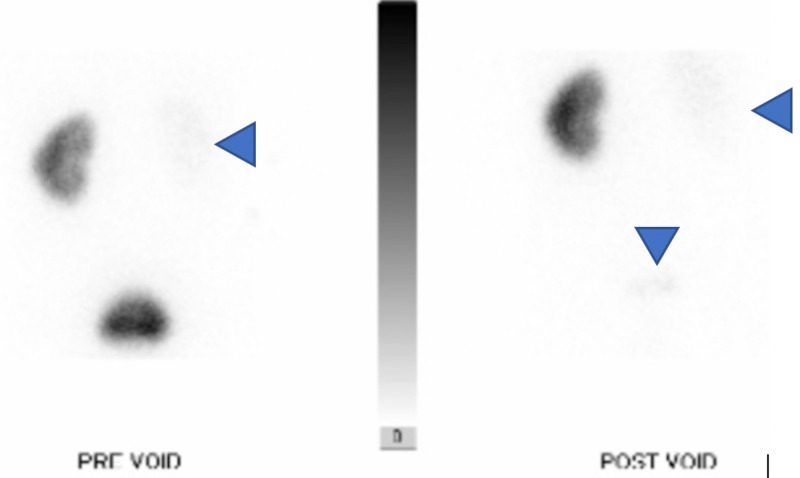
Nuclear medicine MAG3 renal scan Blue arrows show no contrast filling of the left kidney signifying a blockage.

A CT angiography (CTA) of the abdomen and pelvis on his second admission, as seen in Figure 1B, showed an abnormal appearance of the left kidney with some high attenuation prior to the administration of contrast, suggestive of blood. There was essentially no excretion down the ureter from the left kidney. The left kidney had a mottled appearance and surrounding fluid, which was concerning for left-sided pyelonephritis or infarction. Both left renal arteries were patent. As seen in Figure 2, nuclear medicine MAG3 renal scan with Lasix revealed complete obstruction of the left ureter. The split function of the left kidney was 41.9% and that of the right kidney was 58.1%. When correlated to CTA from eight days prior and a CTA from one day prior (Figure 1A, 1B), there was evidence of acute left renal infarction of the lower pole as well as a filling defect in the left renal collecting system consistent with papillary necrosis from left renal infarction causing thrombus in the left collecting system and acute left renal obstruction. Workup for antiphospholipid syndrome was performed, with negative results of all related antibodies. Additionally, the patient showed no abnormalities in COVID-19 testing, lupus anticoagulant studies, antinuclear antibodies (ANA), C3 and C4, prothrombin time (PT), partial thromboplastin time (PTT), and international normalized ratio (INR). A challenging insertion of an 8-French percutaneous nephrostomy was performed by interventional radiology for decompression of the kidney. The patient’s hematuria progressively decreased thereafter, and three days later improved enough for the nephrostomy to be removed.

## Discussion

RPN is a rare renal complication characterized by necrosis of the papillae and inner portions of the renal medulla. RPN may be caused by surgical interventions as well as diseases of vasculature (systemic vasculitides), autoimmunity, hemoglobinopathy (e.g. sickle cell disease [SCD]/trait), pyelonephritis, diabetes mellitus, urinary tract obstruction, tuberculosis, renal vein thrombosis, tubulointerstitial nephritis, cirrhosis of the liver, and transplant rejection, as well as induced by substances such as in the case of analgesic and alcohol abuse [3,4]. RPN typically affects adults (>60 years of age) and is bilateral in 70% of cases [5]. In the more rare events of RPN in the pediatric population, it is more likely to be caused by sickling crises in SCD. Approximately 30% of all cases of RPN are likely due to patients with diabetes mellitus; however, the overall frequency and prevalence is unclear because of underdiagnosis of many asymptomatic cases [6]. Similarly, it is estimated that the incidence of RPN due to SCD is 30-40%, though this etiology may also be underdiagnosed [7]. Regardless, most cases of RPN result in painless microscopic hematuria; therefore, unless symptoms are severe enough to present clinically obvious hematuria or if the ischemic necrosis is large and breaks off into the ureters causing renal colic, these patients generally do not present with complaint to be further investigated. The prognosis is dependent on the etiology and extent of damage, though the prognosis is typically worst in cases complicated by diabetes mellitus [5].

RPN due to renal infarction following vascular interventions can occur as well through dissection of the renal artery itself, renal artery thrombosis, or a cholesterol atheroembolism [8]. Patients with acute renal infarcts following endovascular procedures are generally discovered incidentally on CT abdominal scans since these iatrogenic causes are usually painless; however, some studies report that unilateral abdominal pain, elevated lactate dehydrogenase, hematuria, and renal insufficiency may occur [8]. There have been reports that diagnosis of the renal infarcts has been made up to six days after the endovascular insult [9].

In our patient, the onset of RPN was likely secondary to infarction in the perioperative setting. Pre-procedure imaging in our patient by CTA showed no signs of renal pathology. As is evident in this case, relying on serum creatinine and eGFR (estimated glomerular filtration rate) as sole markers of injury may be misleading. The development of gross hematuria and the drastic changes seen on repeat CTA are consistent with the development of RPN in the postoperative period. Percutaneous nephrostomy was favored over cystoscopy with stenting, given the location of the obstruction. Furthermore, limited workup for hypercoagulability was unremarkable. Given the patient’s heavy smoking history, Buerger’s disease remained in the differential diagnosis.

As mentioned earlier, literature on RPN in this setting is scarcely available. It is well established that ischemia secondary to surgical blood loss or ligation may lead to renal necrosis, and therefore it is certainly theoretically sound to assume this insult can take the form of RPN. In light of this case report, surgeons can be made aware of this potential complication and therefore be better prepared to respond to it.

## Conclusions

RPN should be included in our differential diagnosis of hematuria following endovascular interventions. Our patient developed a clear presentation of renal papillary necrosis in the setting of vascular surgery and was appropriately diagnosed and treated, thus preserving left renal function. Given that many cases of RPN may not present clinically, it may be prudent to carefully monitor renal function and UA in the postoperative period of procedures that may affect renal perfusion.
